# RailFOD23: A dataset for foreign object detection on railroad transmission lines

**DOI:** 10.1038/s41597-024-02918-9

**Published:** 2024-01-16

**Authors:** Zhichao Chen, Jie Yang, Zhicheng Feng, Hao Zhu

**Affiliations:** 1 Department of Electrical Engineering and Automation, Jiangxi University of Science and Technology, Ganzhou, Jiangxi Province 341000 China; 2 Jiangxi Provincial Key Laboratory of Maglev Technology, Ganzhou, Jiangxi Province 341000 China

**Keywords:** Power stations, Electrical and electronic engineering

## Abstract

Artificial intelligence models play a crucial role in monitoring and maintaining railroad infrastructure by analyzing image data of foreign objects on power transmission lines. However, the availability of publicly accessible datasets for railroad foreign objects is limited, and the rarity of anomalies in railroad image data, combined with restricted data sharing, poses challenges for training effective foreign object detection models. In this paper, the aim is to present a new dataset of foreign objects on railroad transmission lines, and evaluating the overall performance of mainstream detection models in this context. Taking a unique approach and leveraging large-scale models such as ChatGPT (Chat Generative Pre-trained Transformer) and text-to-image generation models, we synthesize a series of foreign object data. The dataset includes 14,615 images with 40,541 annotated objects, covering four common foreign objects on railroad power transmission lines. Through empirical research on this dataset, we validate the performance of various baseline models in foreign object detection, providing valuable insights for the monitoring and maintenance of railroad facilities.

## Background & Summary

Railroad power transmission lines play a vital role in modern rail transportation systems, providing a stable and reliable channel for the transmission of power to trains^1,2^. However, when power transmission lines come into contact with conductive materials such as metal foils or metal kite wires, short circuits may be triggered, resulting in loss of train power. Larger insulating lightweight materials may also entangle the pantograph, which in turn affects the normal operation of the train. According to statistics, within 10 days from April 28 to May 7, 2021, Beijing railways experienced a total of 10 contact network failures caused by floating foreign objects due to high winds, resulting in train delays or suspensions. To ensure the safety and reliability of power transmission, it is vital to detect and remove these foreign objects in time.

**Fig. 1:**
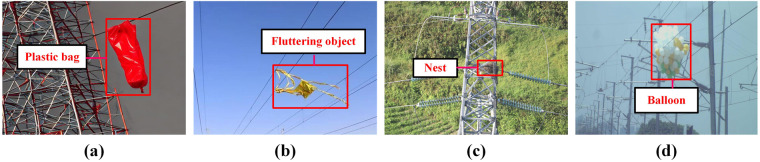
Typical examples of foreign objects intrusion into railroad transmission lines. (**a**) Plastic bags caught in power transmission lines. (**b**) Fluttering objects hanging on power lines. (**c**) Bird’s nest formed on a transmission tower. (**d**) Balloon floating in contact with power transmission line.

Several studies^3,4^ have highlighted the frequent occurrence of power line accidents attributed to plastic bag. These lightweight bags tend to drift close to power transmission lines when the wind is present. Once entangled in these lines, they pose a significant risk, potentially resulting in line failures and power interruptions. Furthermore, these studies^5,6^ emphasize the broader threat posed by fluttering objects to power lines and suggest the need for relevant risk assessment methods. Fluttering objects, such as kites, tethered canvas and fabric materials, exhibit wind-induced vibrations and oscillations, increasing the likelihood of contact with power lines. This contact can lead to line instability and failure, as reported in several instances. Additionally, research^7^ has demonstrated that bird nests can have adverse effects on the insulating properties of power lines. Lastly, there have been reports of electrical accidents caused by balloons^8,9^, primarily due to their uncontrolled floating behavior that brings them into contact with power lines. To summarize, common foreign objects that can be found on railroad transmission lines include: (1) Plastic bags: these lightweight items are easily carried by the wind and can become entangled in transmission lines, posing safety risks (see Fig. 1a). (2) Fluttering objects: objects like kites, tethered canvas, and fabric materials are capable of fluttering or vibrating in the wind, making them a potential threat to power lines (see Fig. 1b). (3) Birds’ nests: bird nests may lead to short circuits, malfunctions, or endanger the birds themselves (see Fig. 1c). (4) Balloons: due to their lightweight and floating characteristics, balloons can cause line failures if they become caught in transmission lines (see Fig. 1d).

In the field of railroad surveillance image analysis, manual inspection of foreign objects on railroad lines is associated with a number of problems, including time-consuming, untimely, and high cost, which pose significant challenges to ensuring the safety and proper operation of railroads. Therefore, automated foreign object detection is widely recognized as an efficient solution in railroad surveillance images^10^. Especially in modern image processing techniques, a lot of research^11–13^ has been conducted in the field of object detection, among which deep learning-based object detection algorithms are the most popular. However, these methods have a huge demand for labeled image data during model training and evaluation. The unique characteristics of railroad monitoring data make data collection exceptionally difficult. First, the presence of foreign objects on railroads is relatively rare. Second, the legal sensitivity of railroad imagery means that there are restrictions and barriers to data collection and sharing. As a result of these limitations, publicly available, large-scale datasets of railroad surveillance images have not yet emerged, limiting the development and application of foreign object detection techniques on railroads.

To summarize, sharing and releasing foreign object datasets for railroad transmission lines is crucial for constructing foreign object detection models. By using this dataset for model training, foreign objects can be detected more efficiently, which will promote the development of artificial intelligence technology in the railway field.

In this paper, we create a comprehensive and diverse dataset named “RailFOD23” specifically designed for foreign object detection on railway transmission lines. RailFOD23 comprises a total of 14,615 high-resolution images, making it a valuable resource for training and evaluating foreign object detection models in the railway domain. The main contributions of this paper are as follows:

1. Creation of the dataset: First, images of anomalous conditions on railroad transmission lines are collected manually. Second, ChatGPT and AIGC (Artificial Intelligence Generated Content) techniques are integrated to successfully generate a large number of anomaly images to overcome the problem of scarcity of anomaly data. Finally, image enhancement methods are used to synthesize the anomalies with normal images to further increase the amount of anomaly data.
2. Public availability of the dataset: The RailFOD23 dataset have been publicly released, making it available to researchers and developers. This will help promote research and innovation in the field of foreign object detection on railroad transmission lines.
3. Technical validation: Including verifying the effectiveness of AIGC-based image generation. As well as benchmarking various mainstream deep learning models to ensure the quality and usability of the dataset. In this way, sufficient experimental data is provided to researchers for reference and further research.

## Related Work

In recent years, deep learning have made significant progress in many fields^14–16^, which is mainly attributed to the ability of deep learning to learn and extract key features from large amounts of data. However, the success of deep learning greatly depends on the quality and quantity of data, and the scarcity of data for some scenarios with extreme data scarcity, such as images of foreign objects on transmission lines, poses a challenge for model training.

Researchers have proposed various solutions to address the issue of data scarcity, with one common approach being based on GANs (Generative Adversarial Networks)^17^. By training a generator and a discriminator on existing data, GANs can generate realistic images, thereby expanding the scale of the dataset. Cooper *et al*.^18^ used a styleGAN network based approach to generate new images with differences by transforming the old images. Similarly, other literatures^19,20^ expanded the collected crack images by GAN networks, which effectively increased the data samples. GAN networks are capable of generating similar images, but due to the need to rely on existing image data and the high similarity of the generated images, it is difficult to fit the real scene. Another common data enhancement method is to utilize image random enhancement technique. Kang and Cha^21^ expanded the data volume from 1203 to 12030 images by using Mix-Up and random cropping methods to synthesize the cracked images. Random cropping is a widely used enhancement in remote sensing image processing^22,23^, and its main purpose is to improve the diversity of images and the generalization ability of models. In addition, some illumination enhancement algorithms^24^ are also widely used to adjust the illumination conditions of the images to generate more variable datasets. Although such enhancement methods are able to expand the dataset to some extent, they still have some limitations, such as the dependence on the existing image base and the limited enhancement effect. Moreover, another feasible method for dataset generation is based on text-to-image synthesis. For instance, cGANs (conditional Generative Adversarial Networks)^25^, VAEs (Variational Auto-Encoders)^26^, and the Stable Diffusion^27^ model have achieved remarkable success in generating high-quality images. Despite the theoretically powerful generative capabilities of these models, there is limited detailed research in the literature regarding their application in dataset generation. The primary obstacle lies in the significant demand for extensive textual descriptions, posing a challenge for researchers to generate sufficiently rich descriptions in practice.

In summary of the above analysis and discussion, this study further explores methods for dataset generation building upon prior research. Firstly, images are manually obtained and processed using advanced tools such as Photoshop to acquire high-quality image data. Secondly, image generation is conducted using ChatGPT and the Stable Diffusion model. Detailed textual descriptions are generated using ChatGPT, and subsequently, images matching these descriptions are generated through the application of the Stable Diffusion model, thereby expanding the dataset. Finally, real railway data from the Railsem19^28^ dataset is combined with random image augmentation and the CDTNet^29^ method to synthesize foreign objects.

## Methods

### Data acquisition

The data acquisition methods in this paper include three parts. The first one is manual synthesis, where realistic synthesis operations are performed by using Photoshop (PS) on the collected railroad scenes. The second is an automatic generation method based on ChatGPT and AIGC. The third is data synthesis based on the Railsem19^28^ dataset.

#### Manual data collection

**Fig. 2:**
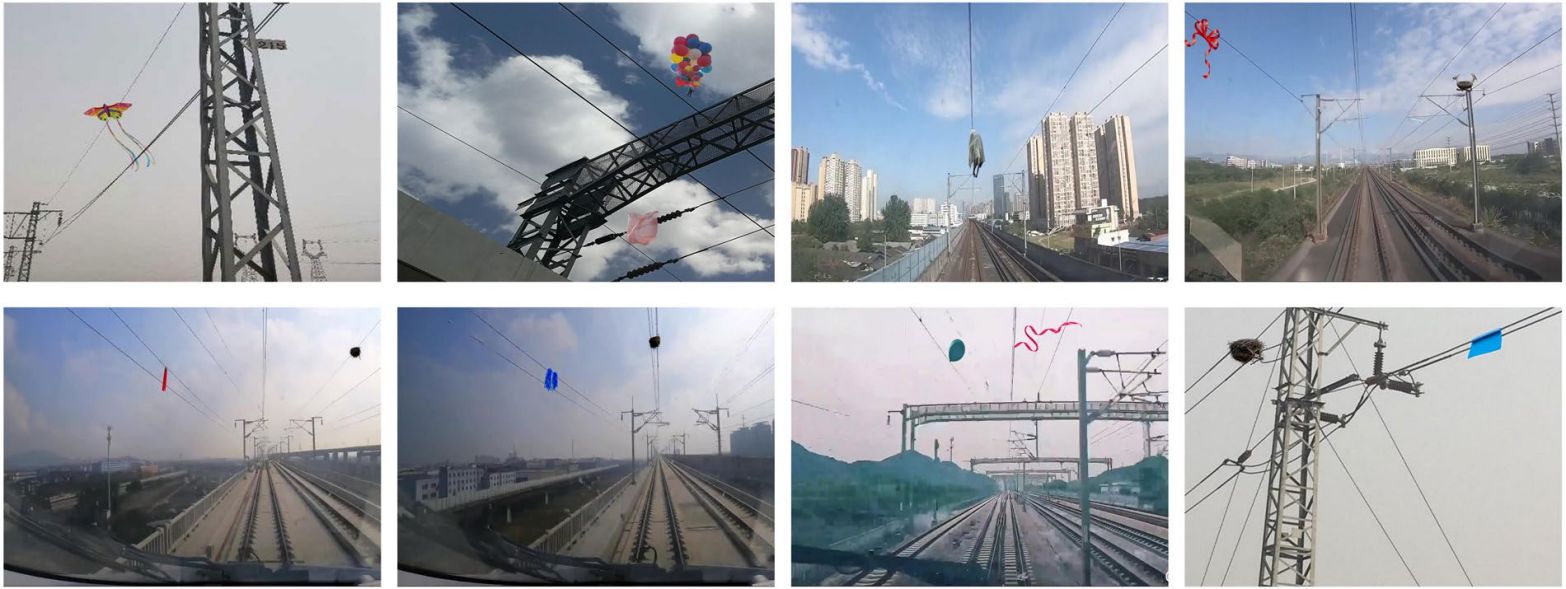
Hand-synthesized image of foreign objects on a transmission line via PS.

About four hundred high-quality images of railroad transmission line scenes were grabbed from the Microbe image library (https://cn.bing.com/images) using Python, and then 412 images of transmission line anomalies were synthesized using Adobe Photoshop (PS) software, as shown in Fig. 2.

#### Image generation based on artificial intelligence generated content

**Fig. 3:**
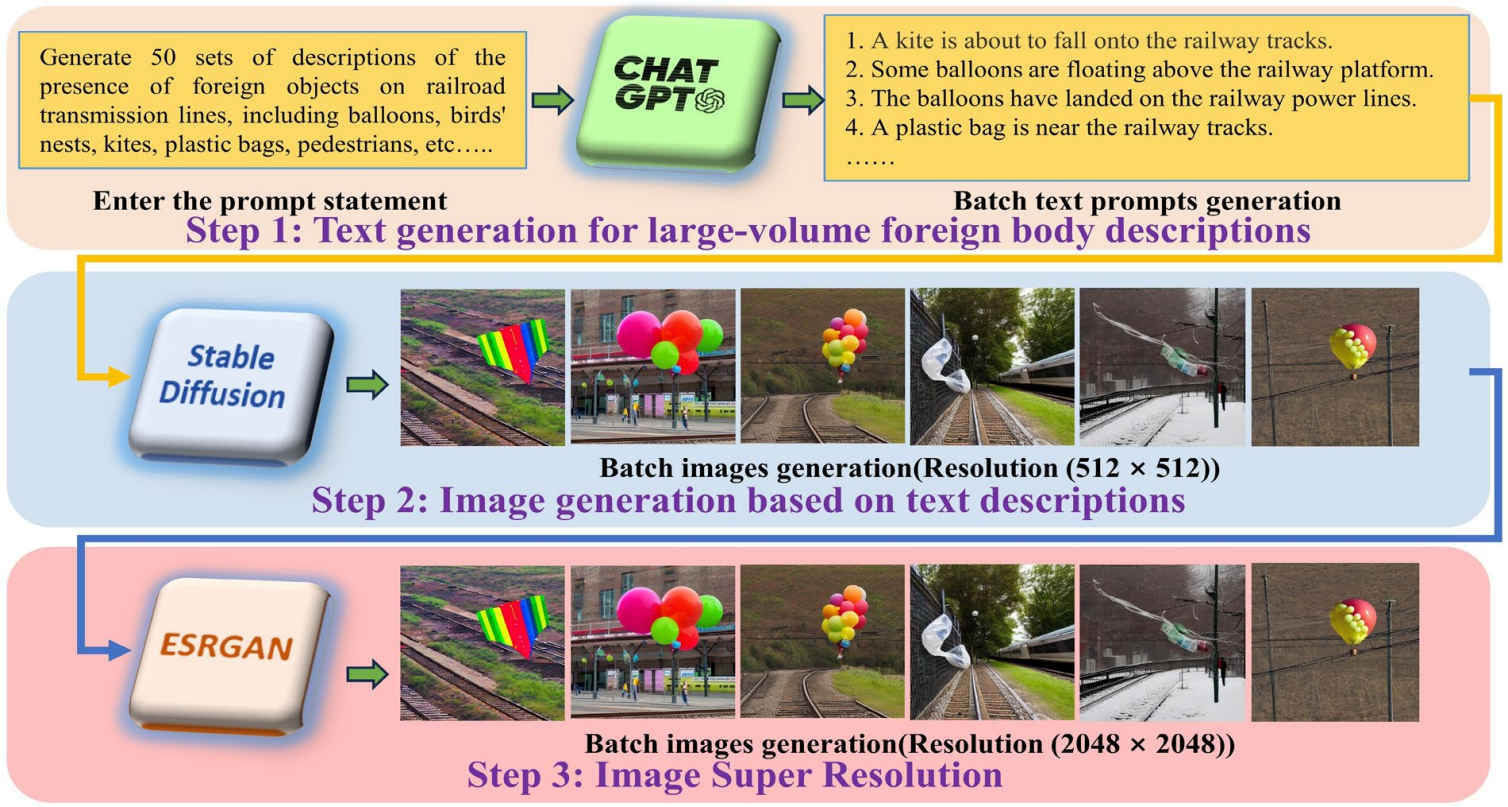
Schematic diagram of generating railroad foreign body images based on ChatGPT and AIGC.

In real-world scenarios, obtaining large numbers of foreign object samples for specific railway scenes is a difficult and time-consuming task. Therefore, to solve the data scarcity problem, a foreign object image generation method based on ChatGPT and AIGC is proposed. As shown in Fig. 3, in order to reduce the training cost, this paper through the current hot three general-purpose model, in turn, batch text generation, image generation, image super-resolution task, the specific details are as follows.

**Step 1:** First of all, it needs to be clear that ChatGPT is a textual dialog model and cannot generate images directly. Note that this paper uses ChatGPT 3.5, which is OPENAI’s current mainstream generalized ChatGPT version (https://chat.openai.com/), without retraining. By inputting fuzzified textual descriptions of railroad scenes, ChatGPT is required to output diverse railroad environments and scenarios. The use of fuzzified descriptions introduces a level of uncertainty, allowing for the inclusion of a diverse range of scenarios. By employing terms like “different” and “possible”, the model is prompted to generate content that captures a spectrum of environmental conditions and potential objects on power lines. For instance, consider the following prompt: “Generate 50 sets of railway power line foreign objects under different weather conditions. The foreign objects possible include items such as balloons, bird nests, kites, plastic bags, and more”. Multiple descriptions are generated using a recursive approach to obtain more diverse textual data, ensuring that AIGC is able to generate diverse images of railroad foreign objects. In the generation of prompts, it is essential to note the following details. First, make sure to use the same session window so that ChatGPT can access contextual information. Secondly, in the course of formulating questions, in addition to describing the sentences needed for generating images of foreign objects on power lines, include the following key prompts: “These sentences are for a subsequent task of generating text-based images”, “Please ensure that each generated sentence is as different as possible from the previous ones”, “The target size of the object can vary”.

**Step 2:** Using the various textual descriptions of the railroad scene from Step 1 as input to the image generation model Stable Diffusion, whose task is to transform these textual descriptions into a composite image of the railroad environment and scene. The model described above is implemented using the mmagic (https://github.com/open-mmlab/mmagic). In this step, it is necessary to manually screen the foreign object images that match the railroad scenario.

**Step 3:** Post-processing and refinement of generated images. After generating a composite image of the railroad environment and scene using stable diffusion, post-processing and quality improvement are necessary. In this paper, the image quality is enhanced using mmagic’s ESRGAN^30^ model, and the resolution can be expanded from the original 512 × 512 to 2048 × 2048.

**Fig. 4:**
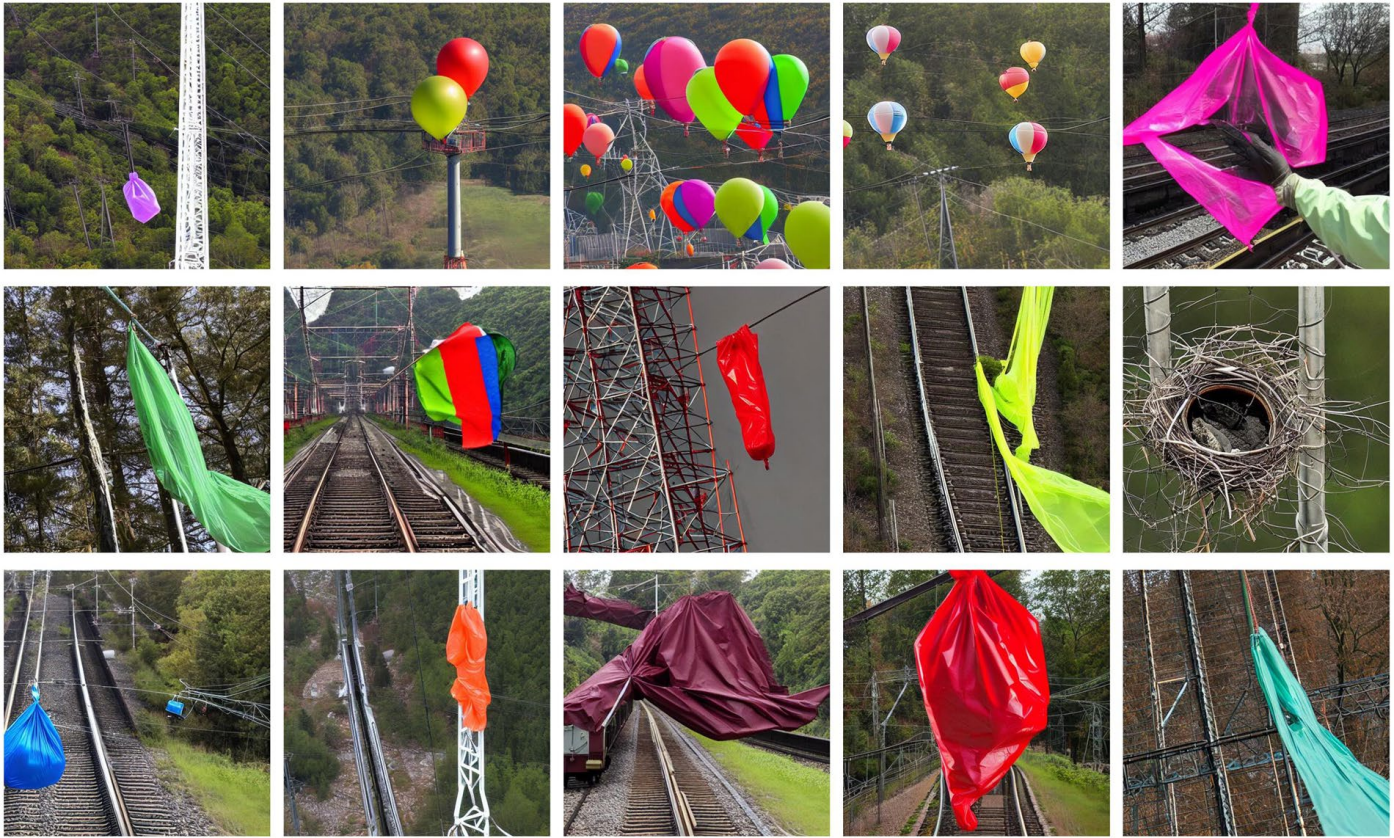
Sample of the final image generated by the proposed method.

By the proposed generation method, a large number of foreign body intrusion data totaling 4000 sheets were obtained, and some samples are shown in Fig. 4.

#### Image synthesis based approach

**Fig. 5:**
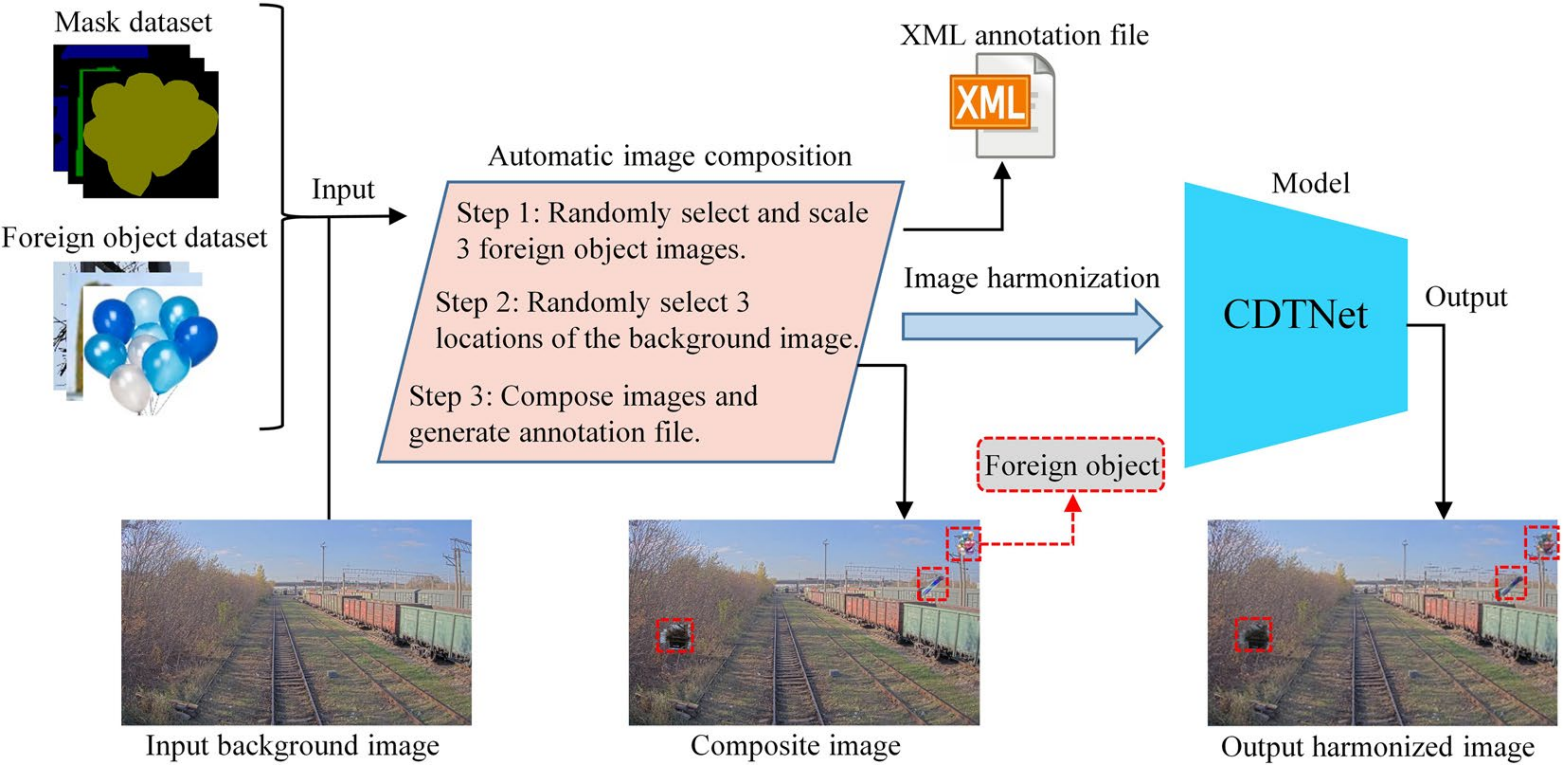
Automatic composition and harmonization of data. Firstly, based on the mask image, the pixels of background image are replaced with the foreign object image. Subsequently, in order to maintain the consistency of image properties, CDTNet is used to achieve image harmonization. Note that the railroad images used are from the Railsem19 dataset.

Manually attaching foreign objects to the background image is an effective method, which allows acquiring foreign object data in a specified scene, but has the following limitations for large-scale images. (1) Manual photoshopping is a complex task, which takes a lot of time and labor. (2) The additional foreign object image and the background may have significant differences, which requires manual effort to adjust the image properties, which further increases the manual workload. (3) The composited foreign object images still need to be labeled. Therefore, an automatic image synthesis method is proposed, which automatically generates labeled files and harmonized images, as shown in Fig. 5.

The workflow of our method is as follows: First, an image dataset of single foreign object is built, and these images size closely fit the foreign object, then a fine foreign object mask dataset is built based on Labelme^31^. Next, three foreign object images are randomly selected from the foreign object dataset, and three coordinates are randomly selected from the background image to attach these foreign objects. Subsequently, the pixels of the background image are replaced based on the corresponding mask image, and an annotation file in XML format is generated. Finally, for the differences between the foreign object and background images, CDTNet^29^ is used to implement image harmonization. In summary, our method can efficiently and automatically generate foreign object images, which can further acquire more usable data.

### Data labeling

**Fig. 6:**
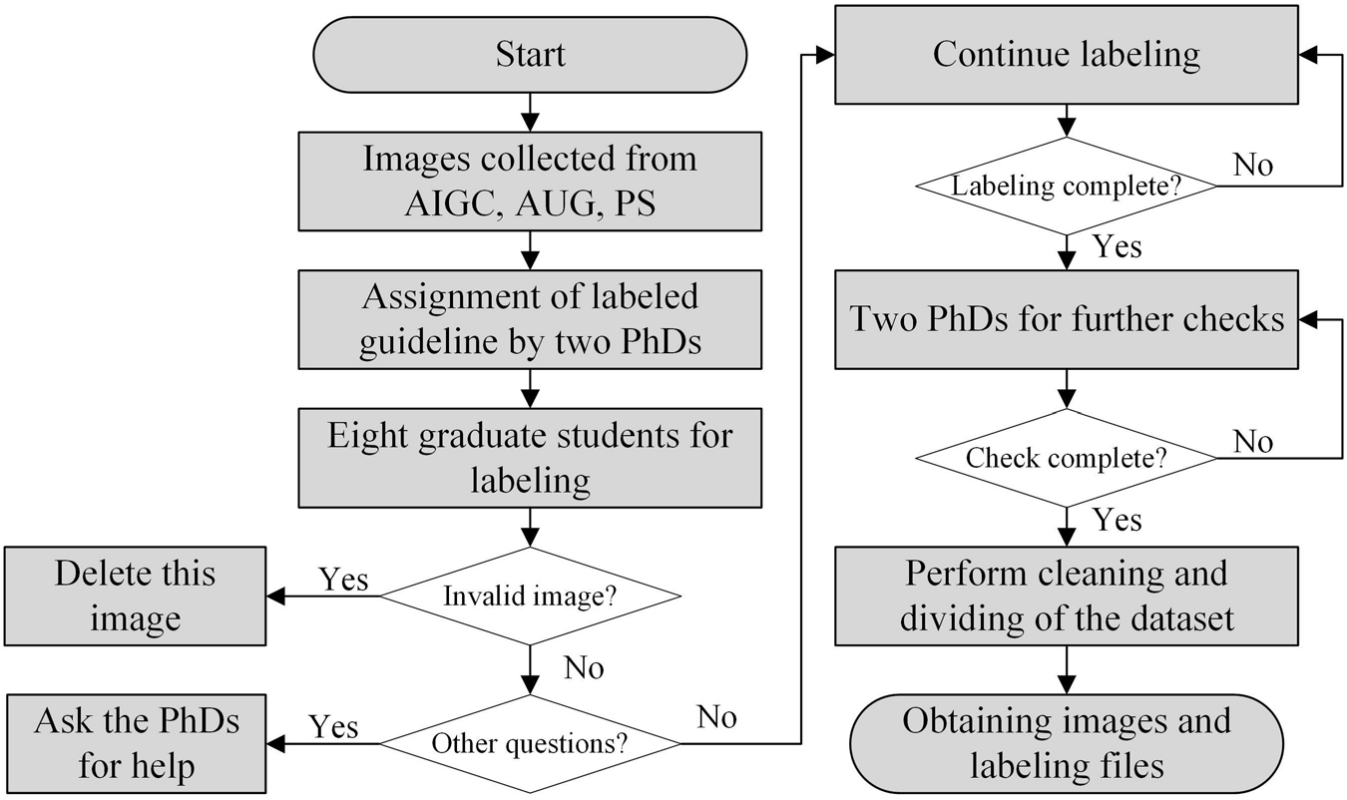
Flowchart of the team to annotate the image data.

The image dataset primarily sources from AIGC, AUG, and PS. The purpose of annotating the dataset is for object detection, the Fig. 6 illustrates the annotation process. In order to ensure consistency and accuracy in annotations, detailed annotation guidelines were formulated by two Ph.D. experts in the field of electrical engineering. The primary annotators consist of eight master’s research students from the group. They may encounter various situations during the annotation process and are capable of providing feedback to the Ph.D. experts. Each annotator initiates the initial annotation of the data after becoming familiar with the annotation guidelines. In cases where annotators encounter images without any discernible objects, they are instructed to delete such images. Upon completion of the annotations, the data undergoes thorough examination by the two Ph.D. experts. If unsatisfactory annotations are identified, problematic images are reannotated by the Ph.D. experts. This iterative process ensures the high quality and consistency of the annotated data. Finally, after the annotation process is concluded, the ultimate image data and corresponding annotation files are obtained.

### Dataset creation and division

#### Dataset preparation

In the preparation phase of the dataset, the COCO data format was used, which has been widely recognized and used in the field of computer vision. First, a random seed of 42 was set to randomly disrupt the data. Next, we divided the entire dataset into a training set and a test set according to an empirical 8:2 ratio. This ratio was chosen to strike a balance between training and evaluating the performance of the model. Note that our dataset is specialized for the object detection task, which means that each sample contains information about the location and class of the object in the image.

#### Evaluation metrics

This paper utilizes several evaluation metrics to comprehensively assess the performance of the dataset and the object detector.

mAP (mean Average Precision): mAP is a key metric to measure the accuracy of the target detection model. It takes into account the precision-recall curves for each category and calculates their average, providing a comprehensive performance assessment of the model across categories.

Number of Parameters: Number of Parameters indicates the number of learnable parameters in the model that need to be trained.

Confusion Matrix: The Confusion Matrix provides detailed information about the model’s classification performance on different categories. It includes the number of true instances, false positive instances, true negative instances, and false negative instances, which helps us to identify the error patterns and strengths of the model on different categories.

## Data Records

The RailFOD23^32^ dataset has been published in zip format on Figshare, as per the data requirements. The entire file occupies approximately 6 GB of disk space, containing an “Images” folder that stores all the images, and an “annotations” folder that contains the.json files with annotations in COCO data format. This dataset is suitable for training object detectors. Users can construct data loading methods based on the COCO format to handle this dataset. By building the appropriate data loading code, researchers can easily utilize the RailFOD23 dataset for training their object detection models. The release of the RailFOD23 dataset provides a valuable resource for researchers and developers to evaluate and improve object detection techniques for foreign object detection on railroad transmission lines. With this dataset, researchers can compare and evaluate different object detection algorithms, driving advancements in this field.

## Technical Validation

### Dataset characteristics

**Fig. 7:**
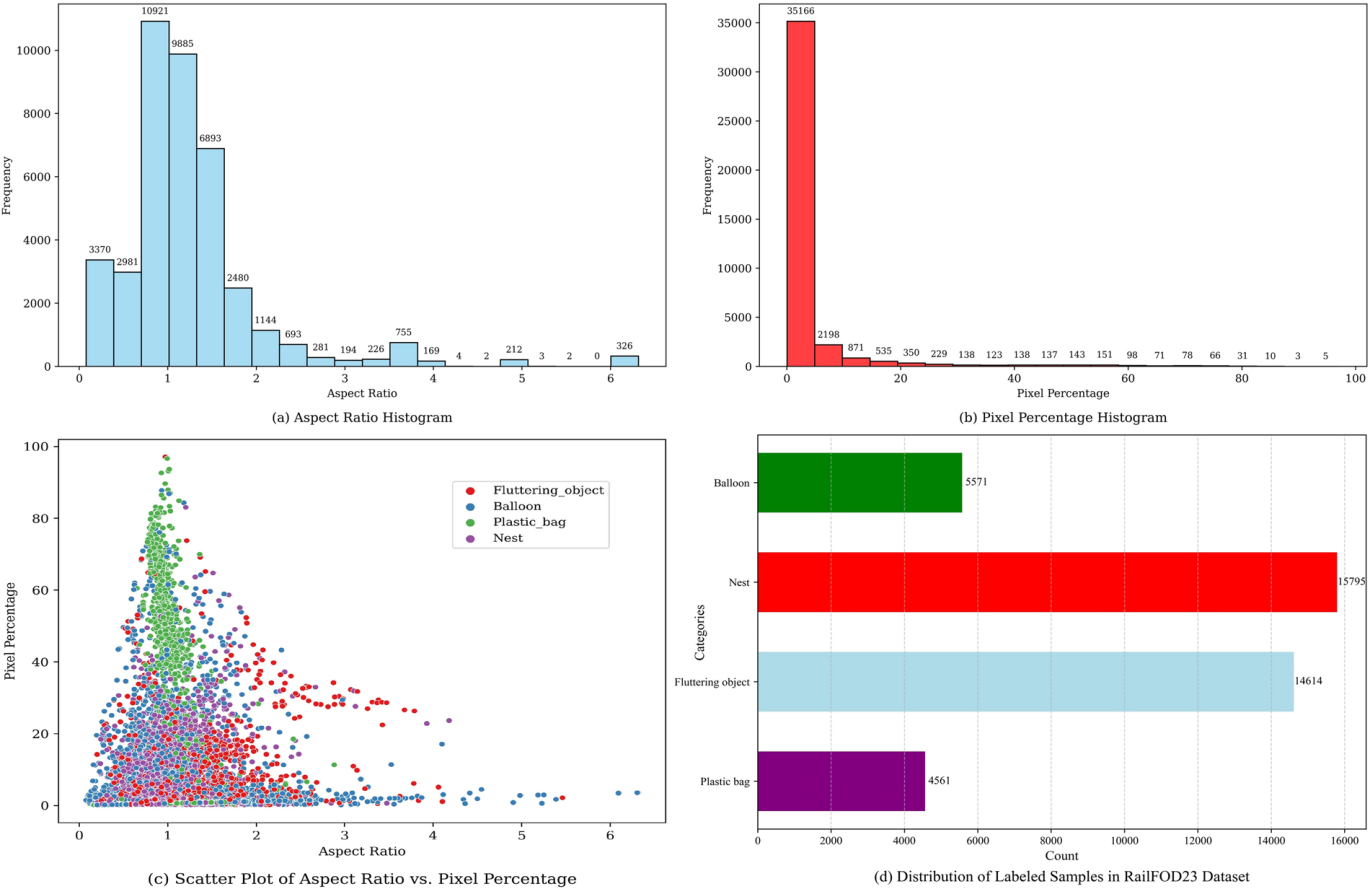
Characterization statistics for the RailFOD23 dataset.

Table 1 provides an overview of the key characteristics of the transmission line foreign body dataset. The dataset is divided into three sub-datasets, AIGC-based, PS-based, and AUG-based, which represent data generated by AIGC generation, PS software generation, and image enhancement methods, respectively. Each sub-dataset contains multiple labeling categories such as plastic bags, fluttering objects, bird’s nests, and balloons. Figure 7 illustrates the characteristic statistics of the dataset, including the aspect ratios of the targets, pixel proportions, while Table 2 provides specific numerical statistics of significance.

**Table 1:** Distribution of labeled samples.

Dataset	Plastic bag	Fluttering object	Nest	Balloon	Total number of labels	Total images	Resolution
AIGC-based	1675	2762	6992	923	12352	5000	2048 × 2048
PS-based	96	279	193	62	630	412	Free
AUG-based	2790	11573	8610	4586	27559	9203	1920 × 1080
Total (RailFOD23)	4561	14614	15795	5571	40541	14615	—

AIGC-based, PS-based, and AUG-based represent data generated by AIGC, PS software, and image enhancement methods on Railsem, respectively.

**Table 2:** Key statistics for the dataset.

Metric	Mean	Standard Deviation	Minimum	Maximum	Median
Aspect Ratio	1.24	0.83	0.0776	6.3148	1.0741
Pixel Percentage	0.0350	0.0954	0.000321	0.9712	0.007656

### Validating images generated by artificial intelligence

When performing validation of the AIGC-generated images, the key objective is to ensure that the generated images have semantic information and can be extracted and recognized by Convolutional Neural Networks (CNNs). To validate the above, the generated images are validated by combining GradCAM (Gradient-weighted Class Activation Mapping) and ImageNet pre-trained weights of the ResNet50^33^. First, the target category *c* to be visualized is selected; the pre-trained network’s supported targets for ours dataset are plastic bags and balloons, corresponding to *c* = {728,417}. Then, the input image is forward propagated through the selected ResNet50 and the target category score *S*_*c*_ = *f*_*θ*_(*x*)_*c*_ is obtained. Calculate the gradient of the target category score *S*_*c*_ with respect to the output feature map *A* of the last convolutional layer of the model. This gradient is denoted as $\frac{\partial {S}_{c}}{\partial A}$. Using the chain rule to compute the gradient:

1 $$\frac{\partial {S}_{c}}{\partial A}=\sum _{k}\frac{\partial {S}_{c}}{\partial {o}_{k}}\frac{\partial {o}_{k}}{\partial A}$$

where *o*_*k*_ is the *k*-th element of the logit vector. Global average pooling of the gradient $\frac{\partial {S}_{c}}{\partial A}$ is computed to obtain weights for each channel. These weights reflect the importance of each channel for the target category *c*.

2 $${\alpha }_{k}=\frac{1}{Z}\sum _{i}\sum _{j}\frac{\partial {S}_{c}}{\partial {A}_{i,j,k}}$$

where *α*_*k*_ is the weight of channel *k*, *A*_*i, j, k*_ is the pixel value on the *k*0. -th channel of the feature map *A*, and *Z* is the size of the pooled region. The weight matrix is multiplied with the output feature map *A* of the last convolution layer using the weights *α*_*k*_ to generate the class activation heat map *L*_*c*_ = *α*_*k*_*A*_*k*_ for the target class *c*.

**Fig. 8:**
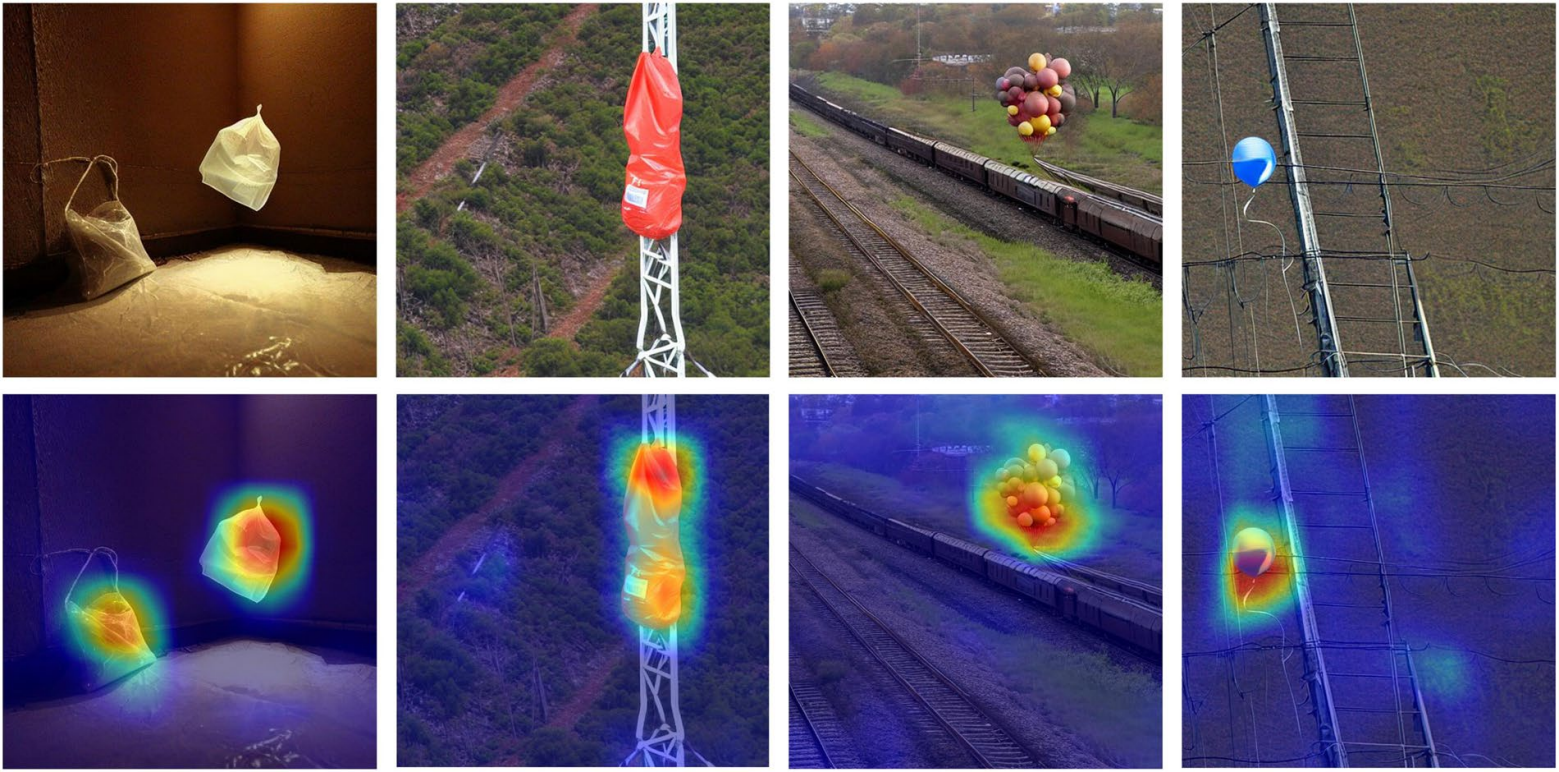
Heat map generated by Grad CAM algorithm.

Finally, we normalized the class activation heat map *L*_*c*_ and superimposed it on the original input image to visualize the convolution neural network’s focus region for the target category *c*. The comparison of the generated heat map with the original map is shown in Fig. 8. In this visualization result, the color shade of the heat map indicates the degree of the network’s focus on the target category, with hotter regions representing the strong focus of the network. It can be seen that for the images generated by AIGC, the CNN is able to efficiently capture these key heat maps, which further proves that the generated dataset is effective.

### Benchmark

To evaluate the performance of different object detection models on the RailFOD23 dataset, we establish a benchmark that includes both one-stage and two-stage object detection methods. The benchmark serves to provide a standardized evaluation criterion for the comparison of the detection performance of various state-of-the-art models.

#### One-stage object detection

The field of one-stage object detection includes several classic algorithms, among which the YOLO^34^ (You Only Look Once) series and the SSD^11^ (Single Shot MultiBox Detector) series are popular. The YOLO series from YOLOv5 to YOLOv8 focuses on lightness and speed, maintaining high performance while aiming for faster inference. SSD is known for its multi-scale nature, which improves performance by adapting to different target sizes with multi-scale anchor boxes. Additionally, there are several one-stage object detection methods available, each offering unique solutions to specific challenges. These methods include RetinaNet^35^, which addresses the issue of positive-negative sample imbalance using focal loss. DETR^36^, which is built upon a transformer architecture and achieves end-to-end object detection and localization through a self-attention mechanism, offering distinct advantages.

In this paper, the above models were selected for training and testing with the aim of demonstrating the feasibility of a transmission line foreign object detection dataset for one-stage object detection.

#### Two-stage object detection

The two-stage object detection approach divides the task into two important phases. First, a candidate region generation network is used to propose regions that may contain targets. Second, a classification and regression network is used to accurately localize and identify the object. In this area, Faster R-CNN^37^ is a pioneering work that introduces Region Proposal Network (RPN), which enables accurate object detection. Libra R-CNN^38^ introduces adaptive positive and negative sample mining to enhance the detection stability and accuracy. In addition, Sparse R-CNN^39^ adopts the sparse attention mechanism to effectively reduce the computational complexity.

#### Comparison of results

To demonstrate the performance of applying advanced object detection techniques on the RailFOD23 dataset to validate the feasibility of deep learning in transmission line foreign object detection, the model fine-tuning technique is utilized to train the mainstream model. The training device for the experiments was a single-card Tesla P100, and the framework used for training was mmdetection^40^. The batch size for training was set to 8, the number of epochs is 40, and the learning rate was 0.001. In addition, pre-training weights were selected for the fine-tuning phase, which were obtained by training on the COCO2017^41^ dataset.

Table 3 shows the test results of the selected mainstream models on the RailFOD23 dataset. In this task, different deep learning models show different levels of performance. First, the Yolo v8-l and Yolo v8-s excel in performance with high mean average precision (mAP), mAP_50_, and mAP_75_. This indicates that these two models have satisfied foreign object detection capabilities for high-precision application scenarios. In addition, the Yolo v8-s strikes a balance between speed and precision and may be an attractive choice for real-world applications. However, there also exist lower performance models, such as Yolo v7-l and Yolo v7-tiny, which perform poorly in detecting foreign objects. Similarly, SSD and RetinaNet (ResNet18) have relatively low performance. DETR (ResNet50) and Faster R-CNN also perform poorly in this task, especially in mAP.

**Table 3:** Performance of the models on the RailFOD23 dataset.

Model	mAP/%	mAP_50_/%	mAP_75_/%	*P*/M
Yolo v8-l^34^	82.9	94.2	88.0	43.66
Yolo v8-s^34^	82.2	93.9	87.2	11.13
Yolo v7-l^34^	31.3	55.5	33.8	36.52
Yolo v7-tiny^34^	18.8	39.4	13.8	6.02
Yolo v6-s^34^	78.7	93.9	85.9	17.19
Yolo v5-s^34^	70.1	91.1	80.8	12.33
Yolo X-s^34^	72.5	91.6	81.7	8.93
SSD^11^	46.3	82.8	48.7	24.14
RetinaNet (ResNet18)^35^	39.6	78.6	34.7	19.83
DETR(ResNet50)^36^	29.1	69.2	18.4	41.55
Faster R-CNN^37^	52.3	89.5	65.2	41.75
Libra R-CNN^38^	68.1	88.2	78.5	41.63

Where mAP_50_ is the mean average precision (mAP) when the intersection over union (IoU) is 50%, mAP_75_ is the mAP when the IoU is 75%, *P* is the number of parameters in the model. M is the unit of millions (10^6^).

**Fig. 9:**
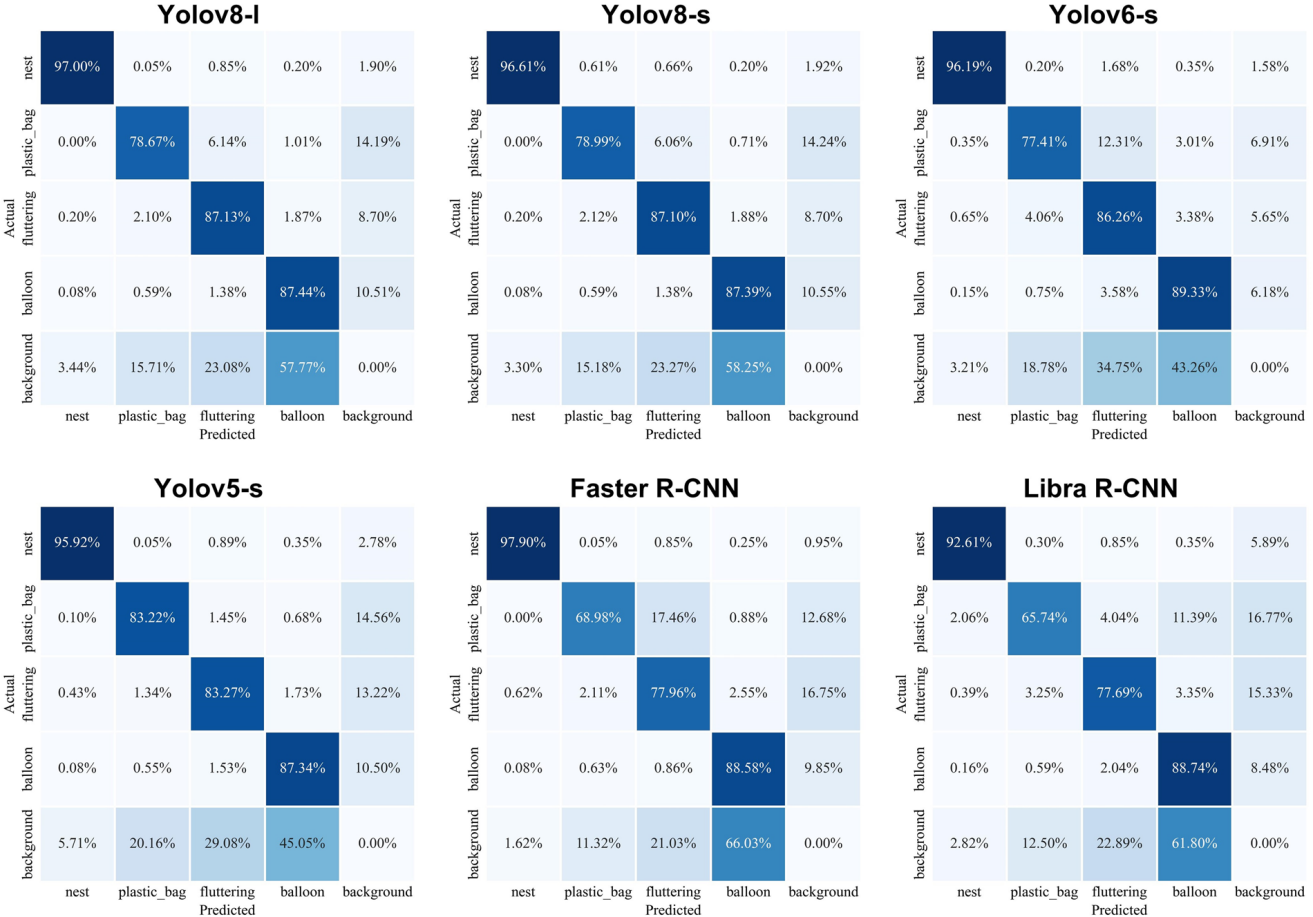
Confusion matrix of the 6 models on the test dataset.

Figure 9 displays the confusion matrices generated by the selected six models on the test dataset. Through these confusion matrices, we can clearly observe the outstanding performance of the Yolo v8 series models. These models excel in the task of detecting foreign objects on transmission lines, as evidenced by the confusion matrices. They exhibit a high number of True Positives (TP), indicating their successful detection of foreign objects.

Table 4 lists the experimental results of the Yolo v8-s model for different combinations of training subsets. These subsets are PS-based, AIGC-based and AUG-based data, respectively. The combination of different training subsets has a significant effect on the performance of the Yolo v8-s model. In particular, the overall performance is improved when AIGC and AUG are fully utilized, which provides effective support for the accuracy of the model under different IoU thresholds.

**Table 4:** Experimental results of training by Yolo v8-s in the case of different combinations of training subsets.

PS-based	AIGC-based	AUG-based	mAP/%	mAP_50_/%	mAP_75_/%
✓			25.1	42.9	28.2
	✓		40.1	60.6	46.1
		✓	64.2	69.2	66.1
✓	✓		45.2	64.3	48.3
✓		✓	71.3	80.5	73.3
	✓	✓	79.6	92.9	85.8
✓	✓	✓	82.2	93.9	87.2

**Fig. 10:**
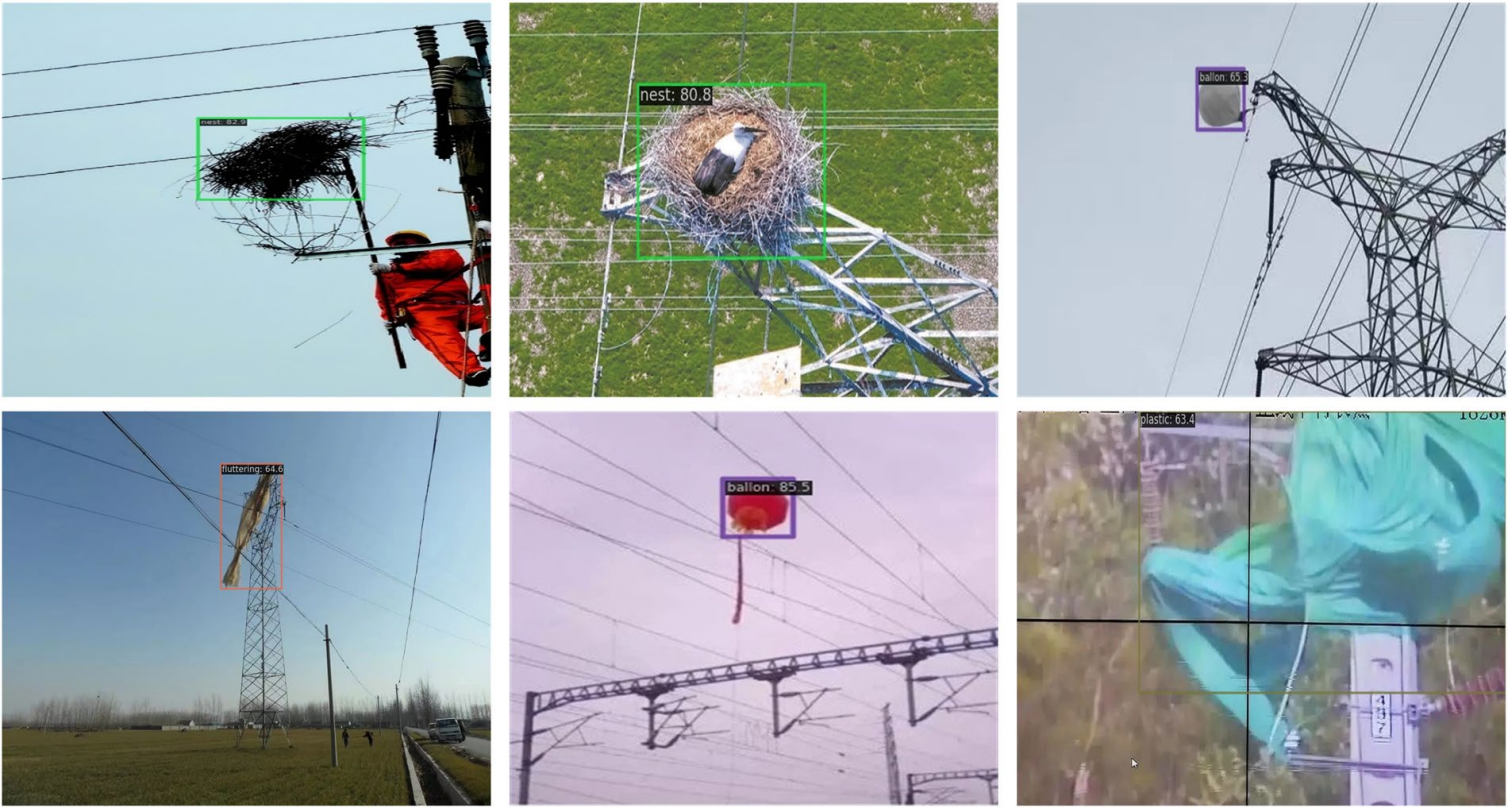
Some real instances of foreign object detection results achieved through YOLO v8-l. The original images are source form http://www.yn.csg.cn/.

Figure 10 illustrates the deployment of YOLO v8-l after training on the dataset mentioned in this paper. The results showcase the detection of foreign objects on six instances of actual transmission line scenes. It can be observed that the model’s predictions exhibit satisfactory accuracy in both object class classification and precise localization within the bounding box.

## Data Availability

We released and shared the code for our data synthesis(https://github.com/CV-Altrai2023/RailFOD23).
